# *In vitro *nuclear interactome of the HIV-1 Tat protein

**DOI:** 10.1186/1742-4690-6-47

**Published:** 2009-05-19

**Authors:** Virginie W Gautier, Lili Gu, Niaobh O'Donoghue, Stephen Pennington, Noreen Sheehy, William W Hall

**Affiliations:** 1 UCD-Centre for Research in Infectious Diseases, School of Medicine and Medical Science, University College Dublin (UCD), Belfield, Dublin 4, Ireland; 2 Mass Spectrometry Resource, UCD-Conway Institute of Biomolecular and Biomedical Research, University College Dublin, (UCD), Belfield, Dublin 4, Ireland

## Abstract

**Background:**

One facet of the complexity underlying the biology of HIV-1 resides not only in its limited number of viral proteins, but in the extensive repertoire of cellular proteins they interact with and their higher-order assembly. HIV-1 encodes the regulatory protein Tat (86–101aa), which is essential for HIV-1 replication and primarily orchestrates HIV-1 provirus transcriptional regulation. Previous studies have demonstrated that Tat function is highly dependent on specific interactions with a range of cellular proteins. However they can only partially account for the intricate molecular mechanisms underlying the dynamics of proviral gene expression. To obtain a comprehensive nuclear interaction map of Tat in T-cells, we have designed a proteomic strategy based on affinity chromatography coupled with mass spectrometry.

**Results:**

Our approach resulted in the identification of a total of 183 candidates as Tat nuclear partners, 90% of which have not been previously characterised. Subsequently we applied *in silico *analysis, to validate and characterise our dataset which revealed that the Tat nuclear interactome exhibits unique signature(s). First, motif composition analysis highlighted that our dataset is enriched for domains mediating protein, RNA and DNA interactions, and helicase and ATPase activities. Secondly, functional classification and network reconstruction clearly depicted Tat as a polyvalent protein adaptor and positioned Tat at the nexus of a densely interconnected interaction network involved in a range of biological processes which included gene expression regulation, RNA biogenesis, chromatin structure, chromosome organisation, DNA replication and nuclear architecture.

**Conclusion:**

We have completed the *in vitro *Tat nuclear interactome and have highlighted its modular network properties and particularly those involved in the coordination of gene expression by Tat. Ultimately, the highly specialised set of molecular interactions identified will provide a framework to further advance our understanding of the mechanisms of HIV-1 proviral gene silencing and activation.

## Background

HIV-1 encodes the nuclear regulatory protein Tat, which is essential for HIV-1 replication and which primarily orchestrates HIV-1 provirus transcriptional regulation. Tat transactivation from the viral promoter (LTR), is highly dependent on complex interactions between Tat, the short leader RNA present in the 5' region of all nascent HIV-1 transcripts, TAR (Trans-activation responsive element), and a number of host cellular proteins [1-4]. The molecular mechanisms whereby HIV-1 gene expression is regulated by Tat occurs at distinct levels. Initially, Tat enhances transcription initiation by promoting the assembly of the RNA polII complex by interacting with various transcription factors [2]. Subsequently, Tat activates elongation via two independent mechanisms: firstly, it enhances the processivity of RNA polII by interacting with elongation factors such as pTEF-b, which phosphorylates RNA polII C-terminal domain, and secondly, by recruiting histone acetyltransferase proteins which modify the chromatin template such as p300/CBP (CREB binding protein) and p300/CBP-associated factor (PCAF) and, as recently described, by interacting with BRM and BRG1, two chromatin remodellers[5-10]. Although the recruitment of these specific cellular factors by Tat to the HIV-1 LTR are crucial for Tat function, they only partially account for the intricate molecular mechanisms underlying the dynamics of proviral gene expression. Furthermore, Tat can be secreted by infected cells and extracellular Tat can exert autocrine or paracrine activities via interactions with cell surface receptors including integrins, CXCR4, CD26, HSPG and LRP[11].

While Tat is a small and compact protein, composed of only 86 or 101 amino acids, sequence and functional analysis reveals that Tat sequence encompasses a unique arrangement of five distinct and contiguous regions including the acidic, cysteine-rich, core, basic and glutamine-rich regions. Furthermore, Tat is subject to post-translational modifications, such as acetylation, methylation, phosphorylation and ubiquitination, thus increasing both the number and diversity of potential interfaces between Tat and cellular proteins [12-14]. Recently, a structural study employing nuclear magnetic resonance (NMR) spectroscopy has described Tat as a "natively unfolded" protein with fast dynamics lacking a well-structured three-dimensional fold. These characteristics would provide Tat the flexibility to interact with numerous cellular partners. Collectively these findings suggest that Tat is a potent, versatile protein suited for multiple interactions and highlights the concept that numerous protein-protein interactions underlie the molecular mechanisms of HIV-1 molecular pathogenesis [15-19].

**Figure 1:**
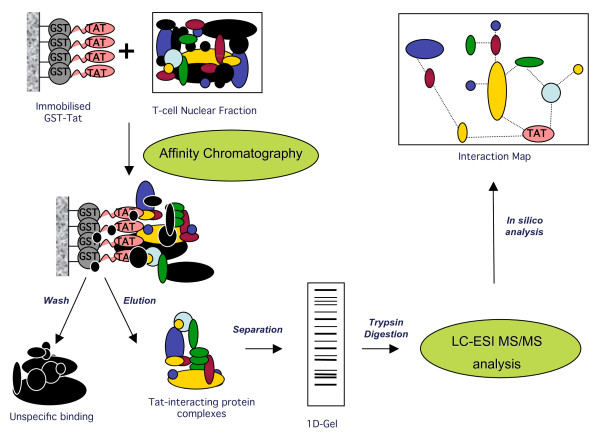
**Overview of our proteomic strategy for isolating and identifying Tat interacting proteins from T-cell nuclear extracts**. Schematic representation of our experimental design based on Affinity Chromatography (AC) coupled with Mass Spectrometry (MS) approach (see text for details).

In this report, we have attempted to further investigate the interplay of Tat with host cell proteins. Specifically, we have designed a proteomic strategy based on affinity chromatography (AC) coupled with mass spectrometry (MS) to purify Tat interacting proteins from T-cell nuclear extracts (Figure 1). Our approach has produced the *in vitro *Tat nuclear interactome, which includes a total of 183 individual nuclear components, most of which have not been previously identified as Tat partners. We subsequently applied *in silico *analysis, to validate our dataset and develop HIV-1 Tat interaction network maps. In this report, we have focused on the description of multi-protein complexes involved in gene expression regulation, which comprised the majority of our dataset and which clearly reflects Tat primary function.

## Results

### Experimental Design

To identify multi-protein complexes associated with HIV-1 Tat, we employed the experimental strategy depicted in Figure 1. Our priority was to ensure a highly sensitive and specific methodology to identify both transient interactions and low-abundance proteins associated with complexes, while ensuring potential contaminants (false positives) remained as low as possible. In this study, we focused on nuclear protein interactions as Tat has been shown to primarily localise in the nucleus. Protein complexes were identified using *in vitro *"pull down" purification employing equivalent amounts of immobilised recombinant GST-Tat (bait) and GST (negative control) proteins, and incubation with T-cell nuclear extracts. Following extensive washes, captured protein complexes were eluted under denaturing conditions (Laemmli buffer) and resolved using a 1D SDS-PAGE gel. For protein identification, GST and GST-Tat interaction profiles within the entire separation range of each SDS-PAGE gel lane were systematically sliced into 2 mm gel pieces and subjected to in-gel tryptic digestion. Peptide mixtures were separated by liquid chromatography (LC) prior to tandem mass spectrometry analysis (MS/MS). The identity of selected proteins was validated by Western Blot (WB) analysis.

### Tat Interaction Profile

**Figure 2:**
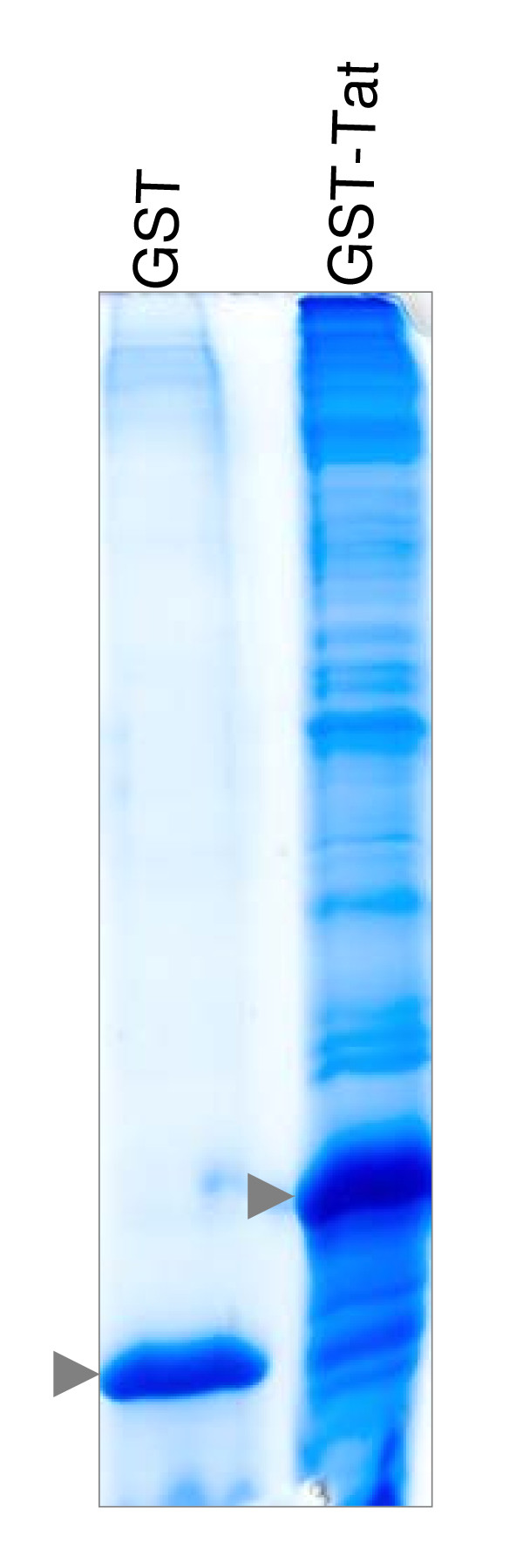
**Interaction profile of Tat associated proteins, isolated from Jurkat nuclear fractions**. T-cell nuclear extracts were incubated with immobilised GST (control) and GST-Tat (Bait). Specifically interacting proteins were subsequently eluted and resolved by SDS-PAGE and stained with Coomassie Blue. The resulting Tat interaction profile is specific and composed of bands of distinct size and intensity, representing putative proteins interacting with Tat. The purified recombinant proteins GST and GST-Tat are indicated by an arrowhead.

Jurkat T-cell nuclear extracts were prepared as described in Materials and Methods and subjected to affinity chromatography (AC) with GST or GST-Tat. GST, GST-Tat and their respective associated proteins were eluted and separated by SDS-PAGE (Figure 2). A total of 164 gel slices from the GST and GST-Tat lanes were processed and the resulting tryptic peptides were analysed by LC-MS/MS. We successfully identified over 250 proteins with sizes ranging from 25 kDa to 400 kDa, which did not interact with GST alone. Proteins were identified with a minimum of two individual peptides (see Table 1 and Additional file 1). In effect, we obtained a moderate to high amino acid sequence coverage by matching tryptic peptides, ranging from 2.5% (MLL) up to 71% (prohibitin), which was inversely correlated with increasing protein size.

**Table 1:** Previously characterised Tat interaction partners.

Symbol	G.O. Process	TurboSEQUEST Score	Coverage %	KD	Accession (GI)	MS/MS Peptide no.	Ref
BRG1	Transcription	258.28	18.9	184529.4	21071056	32 (31 1 0 0 0)	5
INI1	Transcription	40.25	18.30	40666.5	3326993	4 (4 0 0 0 0)	22
BAF170	Transcription	70.26	8.20	132649.7	1549241	7 (7 0 0 0 0)	6
CTIP2	Transcription	70.22	12.8	88420.5	12597635	8 (8 0 0 0 0)	35
ILF2	Transcription	70.35	26.60	44669.4	1082855	11 (10 1 0 0 0)	27, 30
ILF3	Transcription	60.22	16.6	61936.8	9714272	6 (6 0 0 0 0)	27,30
YBX1	Transcription	30.25	16.7	35902.7	27807361	6 (6 0 0 0 0)	21
POLR2A	Transcription	130.28	12	217042.6	7434727	14 (14 0 0 0 0)	24
TAF15	Transcription	20.22	5.4	61520.8	4507353	2 (2 0 0 0 0)	28
ERCC2	Transcription	30.16	5.6	83419.5	296645	3 (3 0 0 0 0)	26, 39
POLR2B	Transcription	40.22	5.1	133810.7	4505941	4 (4 0 0 0 0)	24
BTAF1	Transcription	120.24	11.1	206754.5	27477070	12 (12 0 0 0 0)	28
c1qbp	RNA processing	30.29	23.00	30888.4	338043	5 (5 0 0 0 0)	23
NPM1	RNA processing	126.31	38.8	32582.9	33694244	43 (42 0 1 0 0)	29
EEF1D	Translation	50.27	11.6	71378.2	14043783	5 (5 0 0 0 0)	37
CDC2	Cell cycle	158.31	58.1	34187	30584091	22 (21 1 0 0 0)	33
PPP1CC	Cell cycle	40.29	18.60	36959.8	4506007	4 (4 0 0 0 0)	20
RFC1	DNA replication	50.24	6.2	128174	2136100	5 (5 0 0 0 0)	24
LMNB	nucleus organization	126.22	24.2	66367.7	5031877	13 (12 0 1 0 0)	31, 32
KPNB1	nucleus organization	70.25	11.5	97108.2	19923142	7 (7 0 0 0 0)	36

List of the proteins identified by LC-MS/MS corresponding to known Tat interaction partners. Amino acid coverage (Coverage %), number of MS/MS peptides used for the identification (MS/MS peptide no), TurboSEQUEST score, GenInfo Identifier (GI) for protein and gene ontology (GO) analysis (cellular process) for each identification are indicated.

### Dataset Curation Process

**Figure 3:**
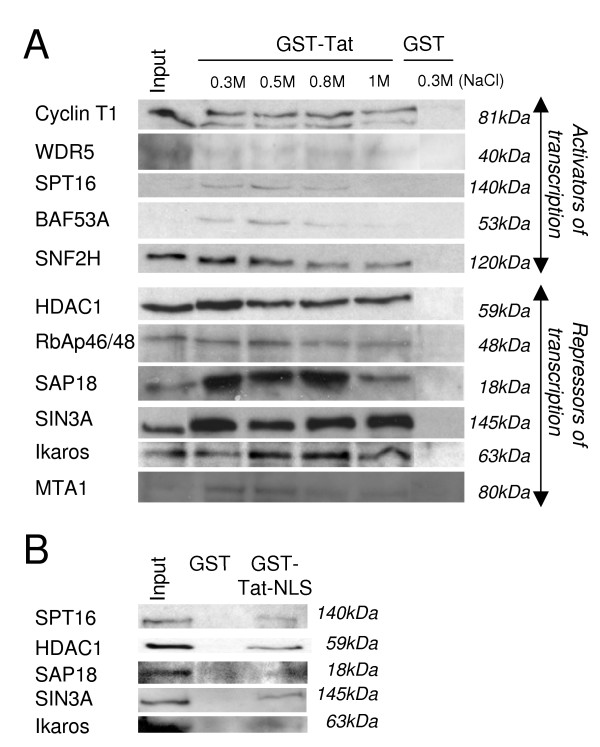
**Validation of the identity of selected proteins interacting with Tat**. A. GST pull-downs were performed with immobilised GST or GST-Tat and Jurkat cell nuclear extracts (150 μg) followed by washes with increasing salt (NaCl) concentration (0.3 M, 0.5 M, 0.8 M and 1 M). Eluates were analysed by WB using the indicated antibodies. B. GST pull-downs were performed with immobilised GST or GST-Tat-NLS and Jurkat cell nuclear extract (150 μg) followed by washes with 300 mM NaCl. Expression levels of each endogenous protein are provided with the Input corresponding to 2 μg of nuclear extracts.

To eliminate potential contaminants from the dataset, we modified our preliminary dataset by retaining proteins known to exist in the nucleus and excluding non-nuclear components such as those associated with mitochondria, cytoskeletal proteins, and common contaminants such as keratin, and ribosomal and histone proteins. The resulting dataset contained 183 candidate proteins that could interact directly or indirectly with HIV-1 Tat. Remarkably, 10% of the selected proteins have been previously identified by other studies, demonstrating the effectiveness and robustness of our approach (Table 1)[5,6,20-38]. The remainder, not previously described, highlighted the potential of our approach to identify new interactions. We subsequently confirmed the identity of 11 proteins identified as new Tat interactors by Western-Blotting analysis (Figure 3). Furthermore, these interactions appear to be robust since some of them, like SIN3A or HDAC1 could tolerate washes containing up to 1 M NaCl (Figure 3A). The specificity of these interactions was further confirmed, employing a Tat-NLS deletion mutant, which still bound with 5 of them (SIN3A, HDAC1, SAP18, Ikaros and SPT16) (Figure 3B). Of note however, our study did not identify certain proteins known to interact with Tat, including cyclin T1, TIP60, P/CAF or BRM[6,8,9,39-41]. However, when we performed GST pull-down with nuclear extract followed by Western-Blot (WB), we could specifically detect the presence of Cyclin T1 in the Tat-eluted fraction, confirming that our recombinant GST-Tat is competent to interact with this well characterised nuclear partner (Figure 3A).

### Dataset Validation Process

In the initial analysis of our dataset, we analysed the domain composition of each protein using CDD, Pfam and Smart databases[42-47]. The 10 most prevalent domains found within the entire dataset are listed in Table 2, where their frequency was compared against their expected frequency derived from the Nuclear Protein Database (NPD) Protein Domains[48,49]. Interestingly, the dataset is highly enriched for interaction domains of RNA (RRM, DEXDc, DSRM) and DNA (HMG and SANT) recognition motifs [50-53]. Other enriched interaction domains are well known to be involved in mediating protein-protein interactions and include the PHD, WD40, RRM or Bromodomain motifs [51,54-56]. Finally, the only enriched domains associated with a catalytic activity were the AAA domain, which is associated with diverse ATP-dependent functions and the DEXDc and HELICc domains, which have a helicase activity[50,57]. Several of these have been previously shown to mediate interactions of cellular proteins with Tat. The protein DICER has been shown to interact with Tat via its DEXDc domain, in an RNA-dependent manner[58]. The WD40 domain of LIS1 also interacts with Tat, and the Bromodomain of P/CAF specifically recognises the acetyl-lysine (K50) in Tat[9,59,60].

**Table 2:** Most representated protein motifs in the Tat interactome dataset.

Motif	N Dataset	% Dataset	% Nucleus
RRM	16	8.4	6.3
HELIC	16	8.4	4.4
DEXDc	16	8.4	3.8
AAA	12	6.3	1.4
BROMO	10	5.2	1.5
HMG	10	5.2	1.9
SANT	8	4.2	1.4
WD40	7	3.7	3.4
PHD	5	2.6	3.2
DSRM	5	2.6	0.7

The number of appearance and percentage of each of the motifs are shown. N Dataset: Number of proteins in Tat interactome dataset possessing motif annotation. % Dataset: percentage of proteins in Tat interactome dataset possessing motif annotation. % Nucleus: percentage of proteins in Nuclear Protein Database (NPD) possessing motif annotation.

When examined individually, these domains are versatile, and occur in a wide variety of proteins. However, collectively, they are frequently found in individual proteins or large complexes associated with key functions in gene expression regulation, and more specifically at the level of chromatin remodelling (HMG, PHD, BROMO, AAA, DEXDc, HELICc, SANT, WD40), gene transcription (RRM, HMG, DSRM, WD40), RNA processing (AAA, RRM, DEXDc, HELICc DSRM) and DNA replication/chromosome structure (AAA, SANT)[50-57,61-67].

Overall, the protein domain analysis exhibited two distinct features: (i) our dataset appears to be specifically tailored to interact with molecules such as RNA, DNA and proteins; (ii) our dataset is highly specialised in gene expression regulation and DNA replication.

**Figure 4:**
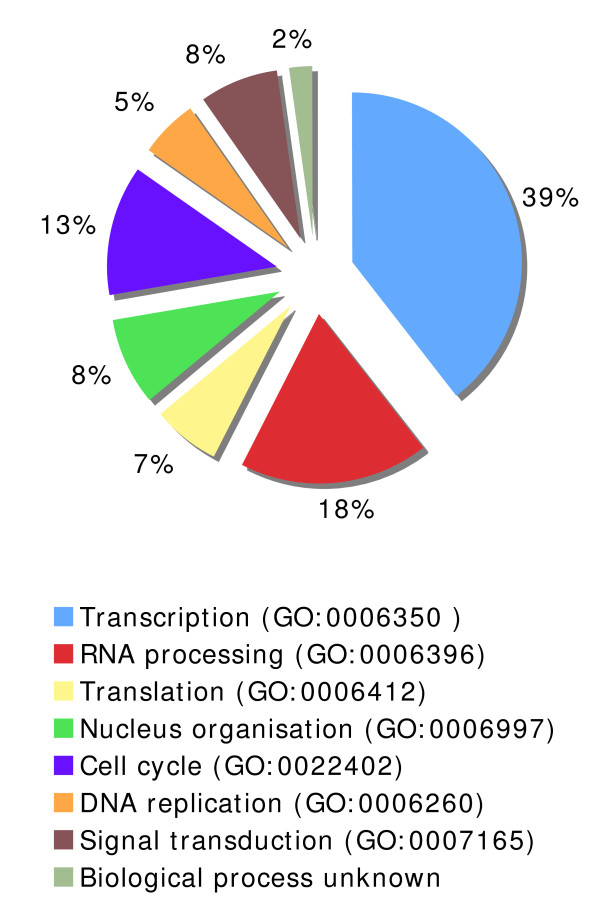
**Functional distribution of Tat interaction dataset**. The assignment of the protein dataset to cellular processes according to G.O. is summarised in the pie chart diagram and the percentage is shown.

To examine functional composition, systematic gene annotation employing the online tool (G.O.) was carried out, and the entire dataset was organised according to the protein involvement in specific biological processes[68,69]. This resulted in the distribution of the proteins over 8 categories, ranging from transcription to DNA replication (Figure 4). Hence, Tat interacts with specific cellular components associated with a range of distinct activities, which may account for the marked pleiotropic activities of the protein. The best represented biological processes include transcription, RNA processing and translation, which collectively accounted for 64% of our dataset. Other major biological processes include cell cycle (13%) and nucleus organisation (8%).

### HIV-1 Tat Interaction Map

#### Construction and mapping of the Tat interaction network

**Figure 5:**
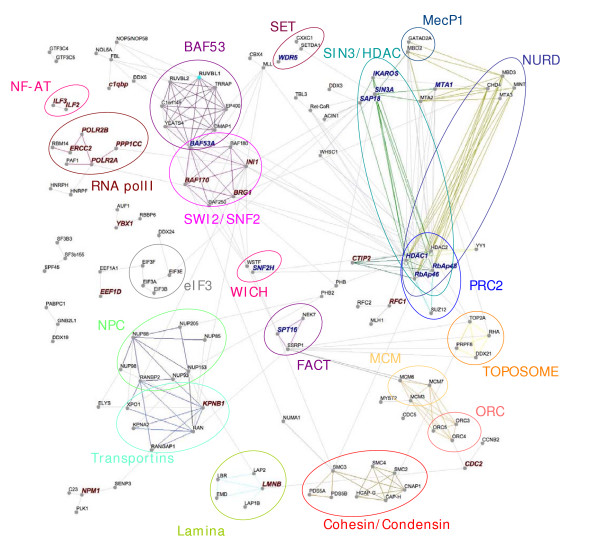
**Tat interaction network**. Here we mapped, using Osprey as a visualization tool, previously established interactions between the individual components of the Tat interaction dataset employing publicly available protein-protein interaction databases (BIND and HPRD) combined with extensive literature search. The network reconstruction of the Tat interactome revealed the higher-order and collective behaviour of the Tat interacting proteins, which compose large but well defined biochemical entities, represented by coloured circles. Edges represent interactions and individual proteins are depicted as nodes. Names in bold and red indicate the previously known Tat interactors and names in bold and blue represent proteins which identity was validated by WB analysis.

While informative, linear analysis of the Tat interaction dataset is inadequate to fully appreciate the higher order organisation of the Tat interactome. As such, we constructed a network representation of the Tat interactome and subjected our dataset to *in silico *interaction analysis and employed Osprey as a visualisation tool[70]. We employed established PPI databases such as BIND and HPRD, complemented by extensive literature searches, to map previously characterised interactions between the candidate proteins and developed a detailed protein interaction network[71-74]. This is depicted in Figure 5[75-233].

#### Global network characteristics

Within the interaction map we included interactions involving a minimum of two proteins. Non-interacting and self-interacting proteins were excluded to simplify the network representation. The resulting network consists of 129 proteins linked by 299 interactions, with an average of 2.31 interactions per protein. Some proteins were highly connected, such as HDAC-1 and -2, which display a total of 23 and 23 interactions, respectively. A striking result of this mapping process was the identification of groups of proteins, which formed distinct and well-connected sub-networks corresponding to previously characterised multi-protein complexes (see below). These well-defined clusters are involved in complementary, consecutive and/or opposite steps of gene expression regulation, epigenetic control, chromosome and nuclear architecture. They include transcriptional repressors, such as SIN3/HDAC, NuRD, PRC2, MeCP1, and activators including SET, FACT, BAF53, SWI2/SNF2 and WICH; chromosome organisation factors including condensin, cohesin, toposome, minichromosome maintenance (MCM), and origin of replication complex (ORC); and nuclear structure including lamina, NPC and transport factors. Interestingly, the complexes could also be shown to be interconnected, which is a reflection of the fact that multiple proteins are shared between distinct complexes, as exemplified by RbAp46 and RbAp48, subunits of the SIN3/HDAC, NuRD and PRC2 complexes. Alternatively, other complexes (such as FACT) remain isolated with protein interactions solely restricted to the members of that complex.

#### Functional modules

Subdivision of the Tat interaction network into functional modules enabled us to gain insights into the functional properties of the multi-protein assembly. The depicted multi-subunit complexes are (i) chromatin modifying factors, which play central roles in the alteration of the structure and composition of chromatin, and are associated with the activation or repression of gene expression; (ii) chromosome organisation factors implicated in mitosis and DNA replication and (iii) nuclear structure components which participate in the nuclear architecture.

##### Activators of transcription

###### SET1

The Set1 histone methyltransferase complex includes, Setd1A, Ash2, CXXC1, RBBP5, WDR5 and Wdr83[234,235]. The SET1 complex mediates the methylation of Lys4 in histone H3, which ultimately results in the activation of transcription. Three components (Setd1A, CXXC1, WDR5) of this complex were identified in the co-eluate by MS/MS and the presence of WDR5 was further confirmed by GST pull-down followed by WB analysis (Figure 3).

###### FACT

The heterodimeric FACT complex (SPT16-SSRP1) has been characterised as an elongation factor, which enables RNA polymerase II to progress through the chromatin template, once transcription has been initiated[236]. FACT acts as a histone chaperone and mediates the disassembly and reassembly of H2A/H2B dimers. Both SPT16 and SSRP1 were identified by AC-MS/MS. We further confirmed the presence of SPT16 in the co-eluate by WB (Figure 3).

###### BAF53, TRRAP/p400

TRRAP/p400 is a chromatin remodeling complex, part of the INO80 family, characterised by a unique subunit composition and the presence of a distinct ATPase[237]. The core of the p400/TRRAP complex, consists of BAF53A, P400, RUVBL1, RUVBL2, TRRAP. These components were identified by our AC-MS/MS approach, and the presence of BAF53A was further validated by WB analysis of GST-pull down products (Figure 3). Additional subunits, including YEATS4, DMAP1 and Eaf6, known to be part of the p400/TRRAP complex, were also identified by our approach. Intriguingly, the TIP60 protein, which has been described in distinct protein complexes harboring p400, BAF53A and TRRAP and is a well characterised interaction partner of Tat, was not detected in the co-eluate[40].

###### SWI2/SNF2

SWI2/SNF2 is another chromatin remodeling complex, part of the SWI/SNF family[238]. Here, we have identified most of the components of BAF (BRG1/BRM, BAF250, BAF170, BAF155, BAF60a, BAF53A, actin and InI) and PBAF (BRG1, BAF180, BAF170, BAF155, BAF60a, BAF53A, actin and InI) complexes except BRM, BAF155 and BAF57. Importantly, BRM, BRG1, InI1 and BAF170 were previously shown to interact with Tat[5,6,22].

###### WICH

The WICH complex, composed of WSTF and SNF2H, is a member of the ISWI-containing chromatin remodeling complexes[239]. In addition to its role in replication, it has been suggested that because of its association with various transcription factors, it may have a role in transcription. Of note, both subunits were identified by AC-MS/MS and the presence of SNF2H in the co-eluate following GST pull-down was confirmed by WB (Figure 3).

##### Repressors of transcription

###### SIN3/HDAC

SIN3/HDAC is composed of SIN3A, SAP30, SAP18, HDAC-1 AND -2 and RbAp46/48 and remarkably, all of these proteins except SAP30 were recovered and identified by our approach. Additionally, the presence of HDAC-1, RbAp46/48, SAP18 and SIN3A in the co-eluate following GST pull-down was confirmed by WB (Figure 3). SIN3/HDAC has been described as a global regulator of transcription; indeed, SIN3A mediates additional interactions with transcription factors and co-repressors, which direct SIN3/HDAC to specific promoters[240]. SIN3A deacetylase activity is mediated by the HDAC-1 and -2 proteins and results in transcriptional repression. Of note, while the presence of HDAC proteins, SIN3A at the level of the HIV-1 LTR has been previously demonstrated, this is the first report showing that Tat interacts with these proteins[241].

###### NuRD

NuRD shares with SIN3/HDAC four individual components which include HDAC-1 AND -2 and RbAp46/48 and additionally contains CDH4/Mi-2, MTA1/2, MBD3, and MINT. NuRD is recruited to target genes via DNA-binding proteins, such as Ikaros identified here by our approach and validated by WB (Figure 3)[242]. In addition to its HDAC activity, NuRD has an ATP-dependent nucleosome activity carried out by CHD4/Mi-2, a chromatin-remodelling ATPase protein, which encompasses a chromodomain of the SWI/SNF family. While we confirmed the presence of MTA1, we failed to detect NuRD in the co-eluate by WB analysis. Interestingly, the presence of MTA1 at the level of the HIV-1 LTR has been previously demonstrated[243].

###### MeCP1

MeCP1, also shares the HDAC-1/-2 and RbAp46/48 subunits and include the methyl-CpG-binding protein MBD2 and p66alpha identified by our approach[84,244-247]. MeCP1 specifically recruits SIN3/HDAC or NuRD to DNA methylation sites recognised by MBD2, which represents an alternative mechanism mediating methylation-dependent transcriptional repression involving histone deacetylation and chromatin remodeling.

###### PRC2

RbAp46/48 are subunits of the Polycomb Repressive Complex 2 (PRC2), which also include EED, EZH1, EZH2, SUZ12. PRC2 can methylate lysine residues (K9 and K27) of histone H3[248]. This ultimately results in the repression of gene expression. Here we have identified SUZ12.

##### Replication and chromosome organisation factors

###### Condensin/Cohesin

The structural maintenance of chromosomes (SMC) proteins form the core of the cohesin and codensin complexes[249]. They are principally involved in chromosome condensation and cohesion and play an essential role into chromatid pairing and chromosome segregation during mitosis. Interestingly, recent studies have described their participation into transcriptional regulation and epigenetic processes. Here we have identified the following condensin I subunits, SMC2, SMC4, CAPG, CAPD2 and CAPH; and the following cohesin subunits: SMC3, PDS5A, and PDS5B to be part of the Tat nuclear interactome.

###### TOPOSOME

The toposome complex consists of the topoisomerase IIα associated with RNA helicase A (RHA), SSRP1, PRP8, hnRNP C and RHII/Gu[142]. This complex is involved in chromosome condensation and segregation and it has been suggested that topoisomerase functions in collaboration with the condensing complex[250]. We have identified topoisomerase IIα, RHA, PRP8 and RHII/Gu as part of our Tat interaction dataset.

###### MCM

The minichromosome maintenance (MCM) proteins are essential for DNA replication and include six members: MCM2–MCM7 which form an heterohexamer complex that binds to DNA replication origins[64,251]. Additional complexes include MCM4/6/7 or MCM3/5. It has been suggested that these complexes play additional cellular roles such as transcriptional regulation and chromatin remodelling. Here we have identified three members of the MCM family, MCM3, MCM6 and MCM7

###### ORC

The origin of replication complex is essential for DNA replication initiation[252]. The binding of the ORC complex marks the origin of replication, where the MCMs proteins are subsequently recruited and uploaded to form the pre-replication complex. However, ORC localisation is not restricted to the origin of replication and has been implicated to a broader spectrum of activities such as silencing and transcriptional regulation, heterochromatin assembly, nucleosome remodeling and chromosome condensation[252].

##### Nuclear structure components

###### NPC and nuclear transport machinery

The Nuclear Pore Complex (NPC) located in the nuclear envelope (NE) is composed of the outer nuclear membrane (ONM) and the inner nuclear membrane (INM), and enables the selective transport of macromolecules in and out of the nucleus[253]. It is composed of over 30 nucleoporins. Here, we have identified several nucleoporins (Nup358, Nup205, Nup153, Nup98, Nup93, Nup88, Nup85) and NPC associated proteins including nuclear transport factors (KPNA2, KPNB1, XPO1, RANGAP1 and RAN) and microfilaments/tubule (TUBB3, TUBBA2, NUMA1 and DNCL1)

###### LAMINA

The nuclear lamina lines the INM and is composed of lamins and NE lamin binding proteins including Nesprin-1 alpha, MAN1, lamina-associated polypeptides-1 and 2 (LAP1, LAP2), emerin and Lamin B receptor[254,255]. The four latter and Lamin B (LMNB) have been identified by our screen as components of the Tat nuclear interactome

These results further substantiate the concept that chromosome architecture, chromatin remodeling, epigenetic control and nuclear organisation constitute pivotal mechanisms in the regulation of HIV-1 provirus gene expression and underscore the diversity of essential biological tasks influenced by Tat interactions.

## Discussion

While considerable efforts have been dedicated to characterise individual proteins or specific macromolecular complexes interacting with Tat, no comprehensive characterisation of the Tat interactome has yet been reported. To place Tat into a wider context of interacting systems and pathways, we systematically analysed protein complexes interacting with Tat within the nucleus, by performing subcellular fractionation followed by AC-MS/MS. This experimental approach is prone to introducing technical artifacts or false positives which can then bias the subsequent analysis. To reduce this, we filtered the raw list of proteins, removed potential contaminants and obtained a final dataset of 183 interaction candidates. Subsequently, we employed computational tools and *in silico *analysis to validate our interaction dataset and to generate a Tat-interaction network representation. This has resulted in the *in vitro *Tat nuclear interactome in Jurkat T-cells.

Our studies have revealed that the Tat nuclear interactome exhibits unique signature(s). The motif composition analysis underlines the enrichment for domains mediating protein, RNA and DNA interactions, which collectively are highly representated in transcription, chromatin remodelling, chromosome structure and RNA processing complexes. We also noted the enrichment of three crucial motifs (HELIC, DEXDc and AAA) associated with helicase and ATPase activities essential for RNA processing, chromatin remodeling and chromosome architecture, which constitute the basis of both DNA replication and gene expression regulation. In support of this, the functional analysis demonstrated that proteins involved in chromatin remodeling, transcription regulation and RNA processing constitute the greater part of our dataset. Finally, the network reconstruction of the Tat nuclear interactome revealed the higher-order and collective behaviour of the Tat-interacting proteins, which compose large but well defined biochemical entities, involved in critical pathways mediating gene expression regulation, chromosome/chromatin structure, and nuclear architecture. Taken together, the remarkable enrichment for essential proteins together with their corresponding macromolecular complexes, and their roles in both activation and repression of gene expression indicate that the described Tat-interactome might act as a modular switch committed to control HIV-1 gene expression.

The presence of numerous, previously identified Tat-interacting partners further validates our dataset. Conversely, critical Tat cellular partners previously identified were not identified by our experimental approach. This could be the result of the level of endogenous expression of these proteins in Jurkat cells and perhaps technical limitations including the following: (i) loss of a fraction of the proteins during the sub-cellular fractionation step; (ii) proteins resistant to trypsin digestion; (iii) proteins not detected by the MS/MS step; (iv) absence of specific Tat post-translational modifications on our recombinant bait, GST-Tat, which is produced by a bacterial expression system. Indeed, Tat interaction with its cellular partner has been shown to be regulated by the acetylation state of its lysines 28, 50 and 51. Interaction of cyclinT1 with Tat requires the acetylation on lysine 28, while acetylation on lysine 50 prevents Tat interaction with Brm but is necessary for its interaction with BRG1 [5-7,256,257]. Nevertheless, despite absence of CyclinT1 from our interaction dataset, we were able to detect it by WB, further establishing our recombinant GST-Tat as a suitable bait.

Our experimental approach does not enable us to distinguish direct from indirect interactions. Furthermore, while we treated our nuclear extract with the Benzonase^® ^nuclease (included as part of the ProteoExtract^® ^Subcellular Proteome Extraction Kit (Calbiochem)), which has both a DNase and an RNase activities, we cannot exclude that some of the observed interactions could be mediated by residual nucleic acids.

To explore the Tat interaction network in detail, we partitioned the interacting candidates into coherent functional modules, based on their reciprocal interactions. Our results further support the recently described role of SNF proteins in Tat-dependent transcription of the provirus[5-7,22,258,259]. Indeed, we have identified BRG1 and the various components of its related complex, SWI2/SNF2. We have also identified for the first time, WICH, an ISWI complex as a candidate Tat interactor.

Other complexes with histone-modifying activities include TRRAP/p400, a histone acetylase, and SET1, a histone methylase, both of which have critical positive effects on transcription. In addition, we isolated the histone chaperone FACT, directly involved in promoting the processivity of RNA polII through the chromatin template, which appears to be of relevance for Tat function in transcription elongation[236].

Importantly, we have provided the first evidence describing a direct or indirect interaction of Tat with cellular proteins, including YY1, HDAC-1/-2, and the components of the SIN3/HDAC and NuRD complexes, previously reported to interact with the integrated HIV-1 LTR. Indeed, earlier studies have identified the presence of HDAC-1 and -2 at the HIV-1 LTR in HeLa cells containing an integrated HIV-1 LTR reporter, as well in latently HIV-1 infected cell lines (ACH2, U1, J-Lat 6.3)[260-266]. They mediate histone deacetylation of nuc-1, the nucleosome positioned immediately downstream of the transcription start site, and are believed to be one of the processes mediating HIV-1 provirus transcriptional silencing throughout the establishment and/or maintenance of HIV-1 latency. In addition, two recent studies have identified the presence of SIN3A and MTA1, a component of the NuRD complex, at the integrated HIV-1 LTR in Jurkat cells, respectively recruited by CBF-1 and CTIP2[241,243]. While various studies have shown that several transcription factors (NF-κB (p50), AP-4, YY-1, c-myc, SP1, CBF-1 and LSF) can recruit HDAC-1 and -2, and SIN3A or MTA1 to the HIV-1 LTR, the molecular mechanism(s) regulating the activity and/or presence of HDAC-1/-2 and their associated complexes at the integrated HIV-1 LTR has not been fully elucidated[241,243,260-266]. The interaction of HIV-1 Tat with the SIN3/HDAC and NuRD complexes further implicates them as potential epigenetic regulators of HIV-1 post-integration latency and suggests how Tat might intersect with epigenetic pathways.

Alternatively, the enzymatic activities of the multi-protein complexes recruited by Tat, such as methylation, acetylation and deacetylation, could be directed to Tat itself and mediate post-translational modifications, as it has been shown previously with PCAF, p300/CBP, SIRT1, or SETDB1/2 [13,14,267,268].

Accumulative evidence has recently described how cellular proteins belonging to the DNA replication and the mitotic chromosome condensation machineries have been shown to carry out additional activities in gene expression regulation and/or silencing by selectively affecting the chromatin/chromosome architecture during the interphase. The identification of Tat interactions with multiple components of the cohesin, condensin, toposome, MCM and ORC complexes provide us with a new prospective on how these pathways might also influence Tat function and HIV-1 provirus expression and silencing.

In addition to its role in regulating the access of specific regulatory factors to the nucleus, the nuclear architecture can affect the genome subnuclear organisation and chromatin structure. In general, there is a strong correlation between the nuclear periphery and heterochromatin establishment and/or maintenance and accordingly gene silencing, while the NPCs have been implicated in preserving euchromatin from such a process[269,270]. More specifically, components of the inner nuclear envelop, lamina and NPC have been described to have an important role in regulating gene expression. LBR, part of our dataset, has been shown to interact with HP1 and consequently regulates heterochromatin formation[271]. Other lamin-associated proteins, such as emerin and Lap2, were also identified as Tat interactors in our studies, have been shown to associate with and sequester a number of transcriptional repressors, including HDACs, BAF, NCoR and beta-catenin[270,272]. The identification of numerous crucial elements involved the nuclear architecture, as Tat interactors, suggest that they could be involved in regulating the transcriptional state of the provirus.

## Conclusion

The results presented here, position the viral regulatory protein at the nexus of a range of interaction networks, which play essential and diverse roles in gene expression, RNA processing, chromatin organisation, chromosome structure and nuclear architecture, and provide the first insights into the modular network properties of the Tat interactome. Ultimately, the HIV-1 Tat rewiring of cellular networks could equip the provirus with a wide repertoire of tools to orchestrate HIV-1 gene expression and confer a remarkable adaptability to a continuously changing cellular environment. Overall, this confirms that Tat transactivation function appears to be the net result of complex interactions with distinct cellular complexes highly specialised in controlling gene expression and more specifically chromosome/chromatin structure. We anticipate that the data presented here will be useful for researchers investigating HIV-1 gene regulation and further studies will delineate the biological significance of these findings.

## Methods

### Cell culture

Jurkat cell line (Clone E6-1) was maintained in RPMI 1640 medium containing 10% fetal calf serum and supplemented with 0.3 mg/L of L-Glutamine (GIBCO) and antibiotics. Nuclear extracts were prepared from Jurkat cells with the ProteoExtract^® ^Subcellular Proteome Extraction Kit (Calbiochem) according to the manufacturers instructions, which include the treatment of the nuclear fraction with Benzonase^® ^nuclease.

### Production of recombinant proteins

GST and GST-Tat (HIV-1 HXB2, 86 amino acids) recombinant proteins were produced in BL21 *E. coli*. and purified with gluthathione-Sepharose beads (Amersham) as described previously[273].

### *In vitro *GST-pull down assays

To create high-density ligand surface, equivalent amounts of purified recombinant GST-Tat (bait) and GST (negative control) proteins were added in excess and immobilised on gluthathione-Sepharose 4 Fast flow (Amersham). The supernatant was discarded and following extensive washes in Binding Buffer (BB) (20 mM Tris, pH 7.4, 300 mM NaCl, 100 mM NaF, 1 mM DTT, 50 mM EDTA, 1% triton X100, 10% glycerol and protease inhibitor cocktail Complete, EDTA-free (Roche)), the beads were incubated with Jurkat cell nuclear extracts (300 μg), rotating at 4°C overnight. Following extensive washes in binding buffer, specifically retained protein complexes were eluted from the resin by incubating the beads in 2× Leamni sample buffer at 95°C for 5 min.

### Gel electrophoresis and Coomassie staining

The eluate was resolved on a 10% SDS-PAGE and the resulting interaction profiles were sliced into 2 mm bands across the entire separation range without bias with respect to size and relatively abundance. A total of 164 gel slices were produced.

### Sample preparation for Mass Spectrometric analysis

1D gel bands were excised from the control and experimental lanes and subjected to in-gel trypsin digestion. Briefly gel bands were washed with sequential additions of ammonium biocarbonate and acetonitrile (ACN) buffers and cysteine residues were reduced and alkylated using DTT and IAA. Samples were digested overnight with 4 ng/μl trypsin at 37°C. Peptides were extracted using 60% ACN, 0.2% TFA buffer and were dried and stored for subsequent MS analysis.

### Protein identification by LC MSMS

LC MS/MS was carried out on a Finnigan LTQ mass spectrometer connected to a Surveyor chromatography system incorporating an autosampler. Tryptic peptides were resuspended in 0.1% formic acid and were separated by means of a modular CapaLC system (Finnigan) connected directly to the source of the LTQ. Each sample was loaded onto a Biobasic C18 Picofrit™ column (100 mm length, 75 μm ID) at a flow rate of 30 nL min^-1^. The samples were then eluted from the C18 Picofrit™ column by an increasing acetonitrile gradient. The mass spectrometer was operated in positive ion mode with a capillary temperature of 200°C, a capillary voltage of 46 V, a tube lens voltage of 140 V and with a potential of 1.8 kV applied to the frit. All data was acquired with the mass spectrometer operating in automatic data dependent switching mode. A zoom scan was performed on the 5 most intense ions to determine charge state prior to MS/MS analysis.

### Data analysis

All MS/MS spectra were sequence database searched using TurboSEQUEST. The MS/MS spectra were searched against the redundant TREMBL database. The following search parameters were used, precursor-ion mass tolerance of 1.5, fragment ion tolerance of 1.0 with methionine oxidation and cysteine carboxyamidomethylation specified as differential modifications and a maximum of 2 missed cleavage sites allowed.

### Western-Blotting analysis

To confirm the MS/MS identification of selected Tat-interacting proteins, we performed Western-Blotting analysis, using BioTrace ™ PVDF (Pall Corporation), on one fifth of the eluate resulting from GST-pull down as described above, but carried out with Jurkat cell nuclear extract (150 μg) and washes in BB including various salt (NaCl) concentrations (300 mM, 500 mM, 800 mM and 1 M). Similarly, GST-pull downs were performed with a Tat-deletion mutant GST-Tat-NLS described elsewhere[273]. The following primary antibodies and their corresponding dilutions were employed: CyclinT1 (H-245) at 1/1000 dilution, mSIN3a (K-20) at 1/5000 dilution, SAP18 (H-130) at 1/5000 dilution, SPT16 (H-300) at 1/1000 dilution (Santa Cruz Biotechnology); and BAF53A at 1/2000 dilution, Ikaros at 1/4000 dilution, HDAC1 at 1/5000 dilution, SNF2H at 1/1000 dilution, WDR51/500 dilution and RbAp46/48 1/1000 dilution (Abcam). the following secondary antibodies (GE Healthcare) and their corresponding dilutions were employed: ECL ™ Anti-mouse IgG at 1/5000 dilution and ECL ™ Anti-rabbit IgG at 1/10000 dilution.

## Competing interests

The authors declare that they have no competing interests.

## Authors' contributions

VG conceived and designed the study, planned and coordinated its execution, conducted experimental procedures, data interpretation and *in silico *analysis, and drafted the manuscript. LG performed GST-pull down/Western blotting experiments. NOD participated in the experimental design and performed LC MS/MS and peptide and protein identification. SP participated in the experimental design and supervised the proteomic analysis. NS participated in the interpretation of results and final editing of the manuscript. WWH supervised the study design, execution, analysis and revised the manuscript critically.

## Supplementary Material

Additional file 1 **List of the 183 components of the Tat nuclear interactome in Jurkat identified by GST pull-down combined with LC-MS/MS**. Amino acid coverage (Coverage%), number of MS/MS peptides used for the identification (MS/MS peptide no), TurboSEQUEST score, Genebank accession number and gene ontology (GO) analysis (cellular process) for each identification are indicated. Click here for file
